# Sandwiched Long-Period Fiber Grating Fabricated by MEMS Process for CO_2_ Gas Detection

**DOI:** 10.3390/mi7030035

**Published:** 2016-02-25

**Authors:** Chao-Wei Wu, Chia-Chin Chiang

**Affiliations:** Department of Mechanical Engineering, National Kaohsiung University of Applied Sciences, Kaohsiung 807, Taiwan; cafa95011@gmail.com

**Keywords:** optical fiber sensor, nanoporous, carbon dioxide

## Abstract

This paper presents an optical fiber gas sensor based on sandwiched long-period fiber grating (SLPFG) that is fabricated via the microelectromechanical systems (MEMS) process and coated with amino silica adsorbent for carbon dioxide (CO_2_) gas sensing. The amine-modified nanoporous silica foams were coated onto the SLPFG for CO_2_ adsorption. To characterize the CO_2_ adsorption of the SLPFG sensor, a gas sensing test was conducted with a mixed gas consisting of 15% CO_2_ and 85% nitrogen at a flow rate of 0.2 L/min. The results showed that the spectra of the SLPFG were varied with the gas flow within 21 min. After that, the transmission spectra of the SLPFG held steady and exhibited no further change. This phenomenon was caused by the adsorption saturation of the amine-modified nanoporous silica foams which were coated onto the SLPFG. During the absorption process, the transmission was increasing by about 11.27 dB (from −23.11 to −11.84 dB), and the increasing rate of transmission was 0.4598 dB/min. Repeatable adsorption and desorption experiment results showed that the SLPFG CO_2_ gas sensor exhibited good repeatability and a short response time. The recovery rate for each cycle was about 85%, and the required recovery time was short. Therefore, elaborated SLPFG gas sensor could potentially be used as a gas sensor for monitoring CO_2_ adsorption in the context of various industrial, agricultural, and household applications.

## 1. Introduction

In the last two decades, interest in the field of optical fiber sensors has developed rapidly. This increasing interest is primarily due to the numerous advantages that optical fiber sensors offer, such as electrically passive operation, immunity to electromagnetic interference (EMI), higher sensitivity, high temperature resistance, multiplexing capabilities, corrosion resistance, and lightness. Due to these advantages, optical fiber sensors have been extensively applied in various fields. Long-period fiber grating (LPFG) sensors can measure most physical quantities, including differing strains, temperatures, pressures, and concentrations [1]. LPFGs consist of periodic refractive index variations, with periods of about 100 μm to 1 mm. LPFGs are very important passive optical components in optical fiber communication and sensing systems. An LPFG is based on the coupling of the core mode to the cladding mode. This coupling phenomenon causes a resonant dip in the spectrum which is very sensitive to external refractive index. As such, LPFGs are very suitable for use as components in sensors. The processes used to manufacture LPFGs include CO_2_ laser writing [2], excimer laser writing [3], arc discharged fabrication [4], the mechanical pressure method [5], and inductively coupled plasma etching process [6] and photoresist method [7]. The LPFG sensor presented in this paper was manufactured using the photoresist method, with the LPFG consisting of etched thin cladding fiber sandwiched between periodic photoresist structures. This particular type of LPFG is called sandwiched long-period fiber grating (SLPFG).

The detection of carbon dioxide (CO_2_) is necessary and important in a wide range of applications, such as the monitoring of global warming, air quality monitoring in mines, indoor and outdoor air quality control, and processing control in the food industry, among others. Global warming, which is caused in part by the gradual increase of CO_2_ in the atmosphere, has become an important and challenging issue. Therefore, finding more effective ways of sensing CO_2_ gas could be of substantial benefit. In 2010, Gouveia *et al.* [8] proposed an indicator free optical fiber sensor for CO_2_ detection. That sensor was comprised of long period grating and a sensing layer based on the acid-basic equilibrium of phenol and its derivative *p*-nitro-phenol. In testing of the sensor, a CO_2_-dependent refractive index change of ~0.05 RIU was observed for CO_2_ concentrations ranging from 10% to 90%. In 2014, Luis Melo *et al.* [9] presented a CO_2_ gas sensor based on LPFG coated with a tactic polystyrene. The sensor had a grating period of 450 µm and a length of 10 mm. In testing of its ability to sense CO_2_, the refractive index of the sensor ranged from 1.3328 to 1.4720. The sensitivity of the coated LPG was 1.23 ± 0.08 pm/% CO_2_, and the sensor resolution was ±4.07% CO_2_. 

In 2015, Karol Wysokiński *et al.* [10] reported an optical fiber CO_2_ gas sensor. The sensor was coated in an organically modified silica sol solution by a dip-coating process, while the sol solution used for preparation of the sensor was made of an ethanol solution of triethoxymethylsilane containing indicator dye. The sensitivity of the sensor, which was defined as a ratio of the light intensities at high and low concentrations was equal to *I*_100%_/*I*_0.1%_ = 8.3 [10]. In this study, we propose an SLPFG sensor coated with amine-modified nanoporous silica foams by the corona method for CO_2_ gas sensing. Coating the sensor with nanoporous silica foams results in changes to the refractive index of the sensor as CO_2_ is captured by the foams. Therefore, we can determine when the test chamber is loaded with CO_2_ gas by observing the changes to the spectra.

## 2. Workingprinciple of the SLPFG Gas Sensor

**Figure 1:**
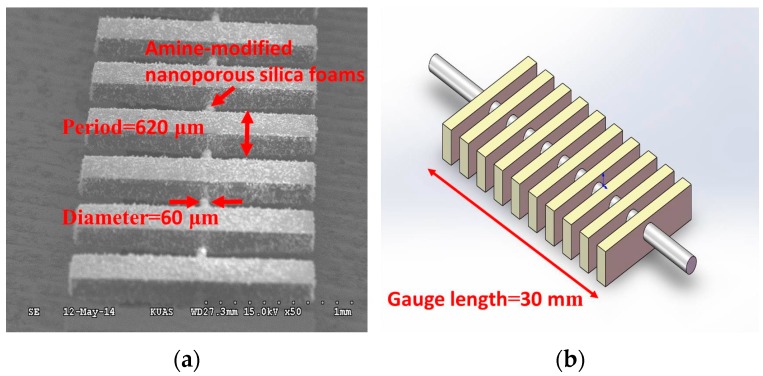
(**a**) Scanning electron microscopy image of the sandwiched long-period fiber grating (SLPFG); (**b**) Schematics of SLPFG.

The SLPFG gas sensor presented herein has grating periods of 620 μm designed for resonance-attenuation wavelengths at 1550 nm. Figure 1 shows the dimensional parameters of the SLPFG, including a 30-mm gauge length, a 620-μm period, and a 60-μm diameter for the etched region.

According to the coupled mode theory [7] for SLPFG, the transmission loss can be calculated by the following Equation (1):

(1) $$T=\cos^{2}(\kappa_{co-cl}^{ac}L)$$

where *T* is the transmission loss of the cladding mode to the core mode, *L* indicates the length of the SLPFG, and $\kappa_{co-cl}^{ac}$ is the AC component of the coupling coefficient between the core and the cladding. The transmission loss of an SLPFG can be deduced from the AC component of the coupling coefficient between the core and the cladding. The transmission loss of a SLPFG is a cosine squared equation. The transmission loss is a function of $\kappa_{co-cl}^{ac}$, which is proportional to the amplitude of changes in the refractive index due to variations in the TEPA-modified powder caused by the absorption of CO_2_ gas. From the above formula, it can be seen that the transmission loss of an LPFG is related to the coupling coefficient and grating length. Therefore, the loss can be altered by the external refractive index. In this study, the TEPA-modified adsorbents coat the optical fiber for CO_2_ gas sensing. As the CO_2_ reacts with the sensing layer, the coupling coefficient and effective refractive index are changed. Therefore, the loss can be changed by the refractive index. From Equation (1), we can measure the CO_2_ gas by monitoring the transmission loss of the SLPFG.

## 3. Materials and Methods

### 3.1. Process and Fabrication of SLPFG

**Figure 2:**
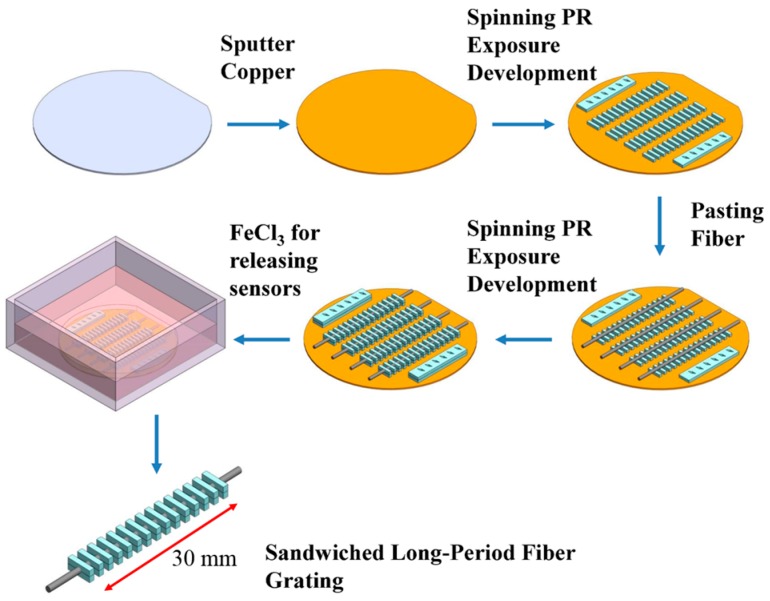
The SLPFG sensor fabrication process.

In this study, grating periods of 620 μm were designed for SLPFG resonance-attenuation wavelengths at 1550 nm. Figure 1 shows a scanning electron microscopy image and schematics of the SLPFG, indicating a 30-mm gauge length, 620-μm period, 60-μm diameter for the etched region. The photolithography microelectromechanical systems (MEMS) process was adopted for producing the SLPFG sensor [7]. The fabrication process is illustrated in Figure 2. The materials used in this process were SU-8 3050 negative photoresist and etched single mode optical fiber. A 4-inch wafer was sputtered with a copper film of approximately 200 nm in thickness, and the single mode optical fiber was etched to 60 μm-diameter with buffered oxide etch (BOE) solution before the process was started. First, spin coater processing was performed to evenly spin-coat photoresist onto the surface of the wafer. Second, a double side mask aligner was used to produce an exposure pattern mask with a 365-nm wavelength ultraviolet (UV) light. Then, the wafer was immersed in a developing solution and rotated by a spinner to remove areas that were not exposed to the UV light. Upon completion of this operation, the designed bottom periodic structure was obtained. Finally, the etched optical fiber (60 μm) was pasted onto the patterned SU-8 structure.

The above procedures were then repeated again to form a structure with a thickness of nearly 125 μm to cover the etched optical fiber. Last of all, the completed fiber grating on the wafer was then immersed in a ferric chloride solution for the releasing process, whereby the photoresist layer was separated from the wafer because the thin copper film sacrificial layer was etched away by the ferric chloride solution.

### 3.2. Preparation of TEPA-Modified Powder

In this paper, the sensitive coating materials used for the gas sensing were synthesized using TEPA (tetraethylenepentamine; C_8_H_23_N_5_) and MSF (mesocellular silica foam), which is a nanoporous silica foam (with cell and window pore diameters of 27.8 and 10.4 nm, respectively), to prepare for the physical impregnation method. After the mixed aqueous amine solution (TEPA) and a stabilizer (titanium isopropoxide) were incorporated into MSF powder and then baked at a temperature of 80 °C, we were able to obtain the required CO_2_ adsorption powder for this experiment. The TEPA-modified MSF powder has a high surface area, a high pore volume, and an orderly porous structure.

### 3.3. Coating the TEPA-Modified MSF Powder Sensing Layer onto the SLPFG Sensor

This study used a surface modification method to tightly bound the TEPA-modified MSF powder to the SLPFG sensor. In order to make TEPA-modified powder that could be tightly adsorbed onto the SLPFG sensor, we used a corona treater with voltage of 10,000–48,000 V to change the hydrophobic material into hydrophilic material, a change that made the powder adhere strongly to the SLPFG sensor. The thickness of TEPA-modified powder sensing layer is about 13.2121 μm. When CO_2_ was subsequently adsorbed onto the SLPFG gas sensor, the effective refractive index of the cladding was changed.

### 3.4. Setup for CO_2_ Gas Sensing Experiment

**Figure 3:**
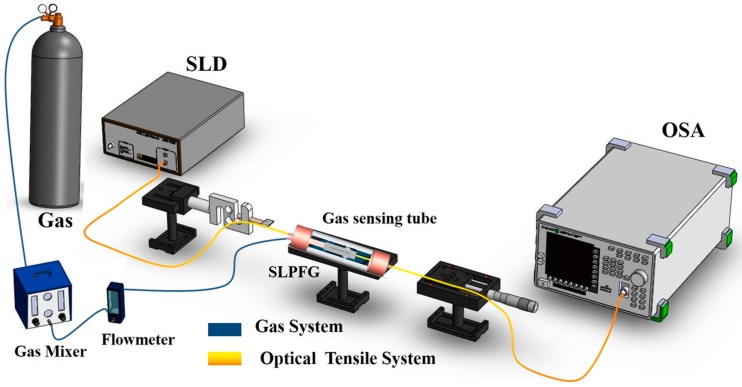
Setup for the CO_2_ gas sensing experiment.

Figure 3 shows the setup for the CO_2_ gas sensing experiment. First, we put the SLPFG sensor into a gas sensing tube which was coated with amine-modified nanoporous silica powder and fixed to both sides of a micro platform and load cell. When the SLPFG sensor was subjected to axial loading, strain fields generated inside the optical fiber formed periodic refractive index changes to produce a long-period grating effect. 

The light source used was an SLD (superluminescent diode), and the light signal was observed by an OSA (optical spectrum analyzer). The gas used in the sensing tests was a mixed gas consisting of 15% CO_2_ and 85% nitrogen that was injected into the gas sensing tube at a flow rate of 0.2 L/min. When the CO_2_ reacted with the TEPA-modified powder sensing layer, the refractive index was changed and then converted into an optical signal for observation.

## 4. Results and Discussion

### 4.1. Results of the CO_2_ Gas Sensing Experiment

**Figure 4:**
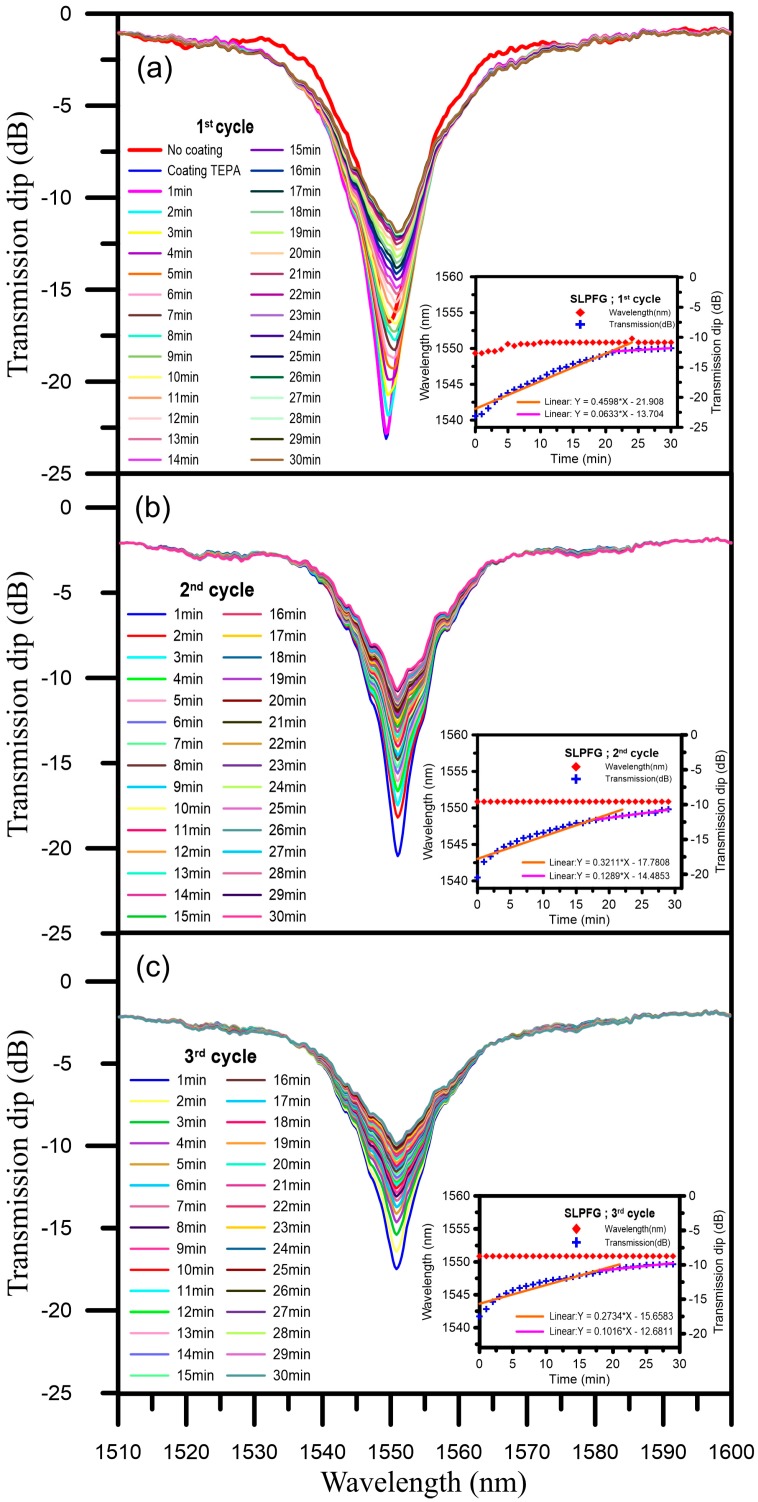
The spectra of the SLPFG during the CO_2_ gas sensing experiment. (**a**) 1st cycle spectra of CO_2_ gas sensing; (**b**) 2nd cycle spectra of CO_2_ gas sensing; (**c**) 3rd cycle spectra of CO_2_ gas sensing. Insets: CO_2_ gas sensing correction graphs for wavelength-time-resonance.

The CO_2_ gas sensing experiments were conducted by using TEPA-modified powder for CO_2_ capture. The experimental results are shown in Figure 4a. When an external loading of 0.392 N was applied to a version of the SLPFG sensor without the coating, the wavelengths of the SLPFG (with a period of 620 μm and fiber diameters of 60 μm) were 1549.5 nm and the transmission loss was −16.74 dB. After the addition of TEPA-modified powder coating to the SLPFG with the surface modification method, the refractive index was changed. This resulted in a decrease in the transmission loss to −23.11 dB, meaning that the transmission loss was decreased by about −6.37 dB. In addition, the resonance attenuation dip was significant, although the wavelength was not shifted. It can be seen that coating the SLPFG with TEPA-modified powder can change the refractive index of the SLPFG to influence the magnitude and position of the attenuation dip. The inset in Figure 4a shows the CO_2_ gas sensing curves of the SLPFG (wavelength/transmission *vs.* time). The total experimental time was 30 min, with monitoring conducted for every minute. The resonant wavelength was not shifted; the transmission loss was increasing by about 11.27 dB (from −23.11 to −11.84 dB).From the inset, it can be seen that the increasing rate of transmission loss was 0.4598 dB/min, the CO_2_ sensor reached saturation within 21 min. After saturation the increasing rate of transmission loss was decreasing to 0.0633 dB/min. The increasing rate of transmission loss of the SLPFG was decreasing significant after saturation. Therefore, the proposed SLPFG gas sensor can successfully monitor CO_2_ absorption.

### 4.2. Repeatable CO_2_ Gas Sensing Adsorption and Desorption Studies

We used a furnace to heat a chamber to 80 °C. The temperature was held constant for 30 min, and then the chamber was cooled to room temperature to allow for desorption of CO_2_. The SLPFG gas sensor transmission was returned to the initial value. The mixed gas consisting of 15% CO_2_ and 85% nitrogen was then injected into the gas sensing tube at a flow rate of 0.2 L/min. Figure 4b shows the transmission spectra of this 2nd CO_2_ gas sensing. The initial transmission value was −20.46 dB. The inset in Figure 4b shows the 2nd CO_2_ gas sensing curves of the SLPFG (wavelength/transmission *vs.* time). The total experimental time was 30 min, with monitoring conducted for every minute. The resonant wavelength was not shifted, the transmission was increasing by about 9.77 dB (from −20.46 to −10.69 dB), and the increasing rate of transmission was 0.3211 dB/min. The CO_2_ sensor reached saturation within 18 min. After saturation the increasing rate of transmission loss was decreasing to 0.1289 dB/min.

**Figure 5:**
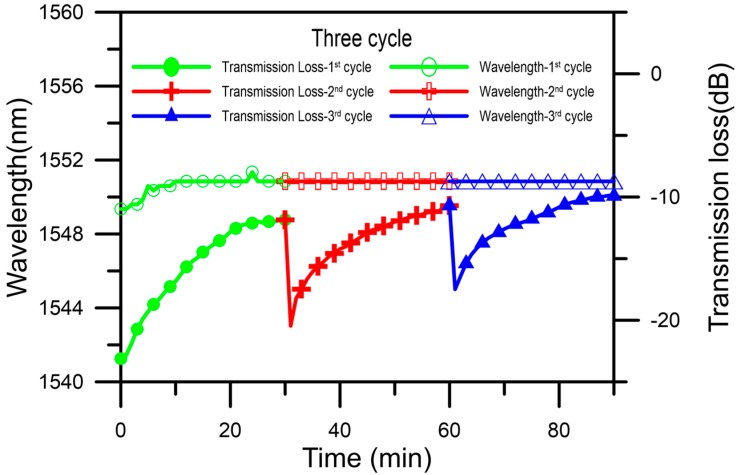
Repeatability of CO_2_ gas sensor.

The 3rd experiment results of the gas sensing are shown in Figure 4c. The initial transmission loss value of the gas sensor was recovered to −17.49 dB. The total experimental time and monitoring time were the same. The resonant wavelength was not shifted, the transmission was increasing by about 7.61 dB (from −17.49 to −9.88 dB), and the increasing rate of transmission was 0.2734 dB/min, the saturation time was 18 min. After saturation the increasing rate of transmission loss was decreasing to 0.1016 dB/min as shown in the inset in Figure 4c. The results of repeatable adsorption and desorption comparison are shown in Figure 5. The recovery rate of the sensing cycle is about 85%. The difference between initial depth and the final depth for the three cycles was caused by degradation of TEPA-modified powder. The phenomenon is caused by the renew process induced degradation of TEPA-modified sensing layer. From the above results, it can be concluded that the SLPFG gas sensor has a good capacity for sensing CO_2_ gas.

## 5. Conclusions

This paper details the manufacturing process for a proposed SLPFG gas sensor utilizing amine-modified nanoporous silica foams as the sensing layer. The results showed that the spectra of the SLPFG changed within 21 min and then reached a steady state. This indicates that the amine-modified nanoporous silica foams were fully saturated with the adsorbed CO_2_ at that time. The transmission loss variation was about 11.27 dB (from −23.11 to −11.84 dB), and the increasing rate of transmission loss was 0.4598 dB/min. The repeatable adsorption and desorption experimental results showed that the recovery rate for each cycle was about 85% and that the required recovery time was short. The results demonstrated that the CO_2_ gas sensor has the advantages of steady performance, simplicity, repeatability, and low cost. Therefore, the SLPFG can be utilized as a CO_2_ gas sensor.
